# Native *Bacillus* Strains Crude Culture Product Exhibits High Biocidal Activities Against Larvae and Adult *Anopheles* Mosquitoes

**DOI:** 10.1155/jotm/8822832

**Published:** 2026-08-27

**Authors:** Tirsit Tibebu, Gemechu Tafa, Teshome Korme, Endale Berhe, Mesfin Getachew Tadesse, Mesfin Tafesse, Musin Kelel

**Affiliations:** ^1^ Department of Biotechnology, Addis Ababa Science and Technology University, Addis Ababa, Ethiopia, aastu.edu.et; ^2^ Biotechnology and Bioprocess Centre of Excellence, Addis Ababa Science and Technology University, Addis Ababa, Ethiopia, aastu.edu.et; ^3^ National Agricultural Biotechnology Research Center, Ethiopian Institute of Agricultural Research, Holeta, Ethiopia, eiar.gov; ^4^ Adami Tulu Pesticide Processing Factory, Ethiopian Chemical Corporation, Adami Tulu, Ethiopia; ^5^ Department of Industrial Chemistry, Addis Ababa Science and Technology University, Addis Ababa, Ethiopia, aastu.edu.et

**Keywords:** *Anopheles* mosquitoes, *Bacillus* spp., mosquitocidal activity, parasporal crystals, SDS-PAGE

## Abstract

*Anopheles* mosquitoes are major vectors of malaria, a disease that continues to cause substantial morbidity and mortality worldwide. *Bacillus*‐based insecticides are widely recognized as effective, environmentally friendly, and sustainable tools for mosquito control. Therefore, this study aimed to isolate native *Bacillus* strains from diverse environmental sources and evaluate their potential for mosquito control. *Bacillus* strains with high biocidal activity were isolated, identified, genetically characterized, and physiologically characterized. The larvicidal and adulticidal activities of culture supernatants and cell pellets were evaluated separately. Eight *Bacillus* strains exhibited strong biocidal activity in their culture supernatants at a concentration equivalent to 8 McFarland units (approximately 2.4 × 10^9^ CFU/mL) within 24 h. The culture supernatants showed significant larvicidal activity, with LC_50_ and LC_95_ values of 0.24 (95% CI: 0.01–0.69) CFU/mL and 1.57 (95% CI: 0.55–4.34) CFU/mL, respectively. The most active strains were identified by biochemical characterization and 16S rRNA gene sequencing. Laboratory bioassays demonstrated that the culture supernatants were effective against both larval and adult *Anopheles* mosquitoes. SDS‐PAGE analysis revealed protein bands ranging from 15 to 130 kDa, including bands within molecular weight ranges commonly associated with putative Cry/Cyt‐like proteins. These findings provide preliminary evidence of proteins that may be associated with the observed mosquitocidal activity. The most active *Bacillus* preparations also showed greater activity than clothianidin under the laboratory conditions tested. Overall, these findings highlight the potential of native *Bacillus* culture supernatants as environmentally friendly candidates for the development of biological mosquito control agents.

## 1. Introduction

Mosquitoes play a significant role in transmitting pathogens to humans and other vertebrates, contributing to substantial morbidity and mortality, largely due to their challenging control [1]. They are the primary transmitters of malaria, one of the leading causes of death worldwide. The most important malaria vectors belong to the subfamily Anophelinae (genus *Anopheles*). Mosquito‐borne diseases account for more than 17% of all infectious diseases, causing approximately 700,000 deaths annually, with 80% of the world’s population at risk of one or more mosquito vector‐borne diseases [2].

Several *Anopheles* mosquito control strategies, including insecticide‐treated bed nets (ITNs), indoor residual spraying (IRS), and larvivorous fish, have been widely used to reduce malaria transmission. Although these interventions have significantly lowered mosquito populations and malaria incidence, their effectiveness is increasingly threatened by the rapid spread of insecticide resistance [3]. In Ethiopia, *Anopheles arabiensis*, the main malaria vector, has developed resistance to multiple insecticide classes, including DDT, pyrethroids (permethrin, deltamethrin, and lambda‐cyhalothrin), and organophosphates (malathion), across different ecological zones [4]. This has reduced the effectiveness of conventional vector control tools and complicated malaria elimination efforts.

The widespread use of chemical insecticides also raises concerns due to their adverse health and environmental effects, including neurological, reproductive, and carcinogenic risks in humans [5]. These challenges have driven the search for safer alternatives. Consequently, entomopathogenic microorganisms are emerging as promising biological control agents for mosquito management, offering targeted action against vectors with reduced environmental impact [6, 7].

Bacteria of the genus *Bacillus*, particularly *Bacillus thuringiensis israelensis* (*Bti*) and *Bacillus sphaericus* (Bs), have been used worldwide for decades as biological products to control mosquito vectors by targeting their larvae [8–10]. *Bacillus thuringiensis serovar israelensis* (*Bti*) de Barjac is the most widely used *Bt* bacterial serotype identified as highly active against *Diptera* larvae [11]. This discovery was followed by the identification of *Lysinibacillus sphaericus* (*L. sphaericus*) Neide strains, which exhibit activity against *Culicidae* larvae [12]. Since the 1980s, products derived from these two bacteria have been regarded as the most successful biological agents for controlling mosquitoes and black‐fly larvae [13]. Both *Bti* and *L. sphaericus* are Gram‐positive, aerobic, spore‐forming, and cosmopolitan bacteria that exhibit strong and selective larvicidal activity against *Diptera*, including genera of major public health importance such as *Aedes*, *Anopheles*, *Culex*, and *Simulium*.

The larvicidal activity of *Bti* and *L. sphaericus* is attributed to the production of crystalline inclusions during their sporulation phase of growth. These crystals consist of protoxins that, once ingested, act on the midgut epithelial cells of mosquito larvae by binding to specific membrane‐bound receptors [14]. The selective mode of action of these toxins is a key feature in meeting the safety requirements for larvicides. Although products based on *Bti* and *L. sphaericus* are powerful and highly selective insecticides, their effectiveness is constrained by optimization challenges, highlighting the need to explore potent, naturally occurring native *Bacillus* species. Accordingly, this study aimed to isolate, identify, and characterize indigenous potent *Bacillus* strains to evaluate their natural biocidal activity against mosquito larvae and adults.

## 2. Materials and Methods

### 2.1. Sample Collection and Bacterial Isolation

These sites are located within the Great Rift Valley of Ethiopia, at a maximum elevation of approximately 1700 m above sea level, and are characterized by hot, arid to cool (subtropical), and humid climates. The mean annual rainfall ranges between 420 and 680 mm, while the mean annual temperature varies from 26°C to 30°C [15].

Samples were collected from different natural resources to maximize bacterial diversity, particularly *Bacillus* species. Sampling was performed during both dry and wet conditions to capture temporal variation in Ethiopia. The dry season sampling was conducted from October 2021 to February 2022, favoring stress‐tolerant, spore‐forming bacteria, while the wet season sampling was carried out from June to September 2022, enhancing microbial growth due to increased moisture. Soil and sludge samples were collected from the Adami‐Tulu insecticide factory in the Oromia region, while water samples were obtained from the Wondo Genet (southern Ethiopia) natural spring and Sodere Lake in Bishoftu, a hot, stagnant spring lake of the Rift Valley mosquito breeding habitat (Supporting File: Figure S1). Dead mosquitoes were collected from Sodere Lake, and additional dead insects were obtained from the Afar region.

A total of 20 environmental samples were collected from different sources, including five soil samples and five sludge samples from the Adami‐Tulu Pesticide Factory, two spring water samples from Wondo Genet, two lake water samples and two dead mosquito samples from Bishoftu Lake, and four dead mosquito samples from the Afar region. Soil samples were collected from a depth of 3–5 cm after removing surface debris, while approximately 10 g of pesticide factory sludge was collected from the waste treatment site. Water samples were collected aseptically in sterile sampling bottles, and dead mosquito samples were collected using sterile forceps and placed in sterile Eppendorf tubes. All samples were transported to the laboratory under cold conditions and stored at 4°C until bacterial isolation.

### 2.2. Bacterial Isolation and Culture

One gram of each soil and pesticide factory sludge sample was suspended in 10 mL of sterile distilled water and thoroughly homogenized. For water samples, 10 mL of each sample was aseptically transferred into sterile test tubes. Dead mosquito samples were surface‐sterilized by rinsing twice with 70% ethanol, followed by two rinses with sterile distilled water. The mosquitoes were then macerated in 2.0 mL of sterile saline (0.5% *w*/*v*) using a sterile mortar and pestle and homogenized with a sterile glass rod. All prepared sample suspensions were heat‐treated at 80°C for 10 min to enrich for spore‐forming bacteria, after which six serial tenfold dilutions were prepared in sterile peptone water. Aliquots (100 μL) from the appropriate dilutions were spread onto nutrient agar containing yeast extract (3 g/L), peptone (5 g/L), sodium chloride (5 g/L), and agar (15 g/L) and incubated at 30°C for 96 h. Colonies exhibiting morphological features typical of the genus *Bacillus* were selected for further purification and characterization.

### 2.3. Bacterial Strain Characterization

Colony morphology was evaluated based on colony size, shape, texture, elevation, and color. Cell morphology and Gram reaction were examined following Gram staining using a light microscope. Endospore formation was assessed using the malachite green staining method. Biochemical characterization was performed using the indole, catalase, and starch hydrolysis tests.

### 2.4. Mosquito Rearing and Maintenance

Laboratory strains of *Anopheles* mosquitoes were obtained from the Ethiopian Public Health Institute (EPHI) insectary. In addition, field‐collected *Anopheles* mosquitoes were collected from Sodere and the Afar Rift Valley hot spring area. The mosquitoes were maintained at Addis Ababa Science and Technology University (AASTU), Ethiopia, under standardized conditions as described by Baeshen et al. [16]. Larvae were also obtained from EPHI, and approximately 200 newly hatched first‐instar larvae were transferred into plastic trays (34 × 24 cm), each containing 1 L of deionized water. Pupae were collected daily, placed in flasks containing distilled water, and transferred to rearing cages until adult emergence [16]. Larvae were fed Tetramin fish food. *Anopheles* mosquitoes were maintained at 26 ± 2°C, 80% relative humidity, and a 12:12 h light–dark photoperiod. Adult mosquitoes (males and females maintained together) were provided with a 10% sucrose solution and blood‐fed using an artificial feeder with pig blood. All experiments were conducted in accordance with relevant regulations and followed the World Health Organization [17] guidelines.

### 2.5. Preliminary Mosquito Larval Bioassays

Overnight cultures of each *Bacillus* isolate were first prepared to refresh and activate cells. The refreshed cultures were then inoculated into fresh medium and incubated at 30°C with shaking at 180 rpm for 5 days to promote sporulation. After incubation, bacterial suspensions were prepared in 10 mL of sterile distilled water and standardized to approximately 1 × 10^8^ CFU/mL using the McFarland turbidity standard. Preliminary larvicidal activity was evaluated by exposing 25 third‐instar *Anopheles* larvae to each bacterial suspension in 30 mL plastic cups containing 20 mL of distilled water. Larval mortality was recorded at 12, 24, and 48 h after exposure by counting dead larvae. Isolates causing 100% larval mortality during preliminary screening were selected for further characterization and detailed larvicidal bioassays.

### 2.6. Preparation of Bacterial Cells and Culture Supernatant

Selected *Bacillus* isolates were inoculated into 5 mL of nutrient broth medium and incubated at 30°C with shaking at 180 rpm for 5 days to promote bacterial growth and sporulation. After incubation, the cultures were centrifuged at 10,000 × g for 10 min to separate the bacterial pellet from the culture supernatant. The supernatant was collected, filtered through a sterile membrane filter, and transferred into sterile Falcon tubes for subsequent bioassays. The bacterial pellet was washed with phosphate‐buffered saline (PBS) and resuspended for evaluation in subsequent bioassays.

### 2.7. Larvicidal Assay Using Culture Supernatant and Bacterial Pellet

The larvicidal activities of the culture supernatant (SUP), bacterial pellet (PEL), and their combination (SUP + PEL) were evaluated using the isolates selected from the preliminary screening. Bacterial pellet suspensions were standardized to 2, 4, and 8 McFarland units, corresponding approximately to 6 × 10^8^, 1.2 × 10^9^, and 2.4 × 10^9^ CFU/mL, respectively. The culture supernatant was used at equivalent volumes obtained from the corresponding bacterial cultures (Supporting file: Figure S2). For each treatment, 10 third‐instar *Anopheles* larvae were exposed in Petri dishes, and each assay was performed in triplicate. Treatments consisted of (i) SUP, 1 mL of culture supernatant + 10 mL of distilled water; (ii) PEL, 1 mL of bacterial pellet suspension + 10 mL of distilled water; and (iii) SUP + PEL, 0.5 mL of culture supernatant + 0.5 mL of bacterial pellet suspension + 10 mL of distilled water. A negative control containing distilled water without bacterial material was included in each experiment. All mixtures were vortexed for 1 min before larval exposure. Mortality was recorded at 12, 24, and 48 h postexposure (Supporting file: Figure S3). Percentage mortality was calculated as the number of dead larvae divided by the total number of exposed larvae × 100 (Supporting file: Table S1). Mean mortality (%) ± standard error (SE) was calculated from three independent biological replicates and used for subsequent statistical analyses.

### 2.8. 16S rRNA Gene Sequence Analysis

Genomic DNA was extracted from pure bacterial cultures exhibiting strong mosquitocidal activity using a DNeasy Blood & Tissue Kit (QIAGEN, Valencia, CA, USA) according to the manufacturer’s instructions. The primer pair rD1 and fD1 [18] was used to amplify an approximately 1500‐bp fragment of the 16S rRNA gene. PCR amplification was performed under the following conditions: an initial denaturation at 95°C for 5 min; 35 cycles of denaturation at 95°C for 30 s, annealing at 55°C for 1 min, and extension at 72°C for 30 s, followed by a final extension at 72°C for 5 min. The amplified PCR products were subsequently sequenced using the same primers on an ABI 3730XL Genetic Analyzer: Fd1 (AGAGTTTGATCCTGGCTCAG) and Rd1 (AAGGAGGTGATCCAGCC).

### 2.9. Phylogenetic Analysis

The 16S rRNA gene sequences of bacterial isolates were compared with reference sequences available in the NCBI GenBank database (National Center for Biotechnology Information) using the nucleotide Basic Local Alignment Search Tool (BLASTN) to determine their closest phylogenetic relatives. The edited sequences were aligned with closely related reference sequences using MEGA 12. Phylogenetic trees were constructed using the neighbor‐joining method with 1000 bootstrap replicates to evaluate the reliability of the inferred relationships among the bacterial isolates.

### 2.10. Physiological Characterization of the Molecularly Identified Bacterial Isolates

The molecularly identified bacterial isolates were evaluated under different environmental conditions to determine their growth characteristics. The isolates were cultured under varying pH levels (3, 3.5, 4, 4.5, and 5), NaCl concentrations (2%, 4%, 6%, 8%, and 10%), and incubation temperatures (20°C, 25°C, 35°C, and 45°C). Bacterial growth in broth culture was assessed by measuring the optical density at 600 nm (OD_600_) after 24 h of incubation. The growth responses under each condition were used to determine the optimum pH, salinity, and temperature for each isolate.

### 2.11. Adult Mosquitocidal Assay

Adult toxicity bioassays were conducted using five‐day‐old *Anopheles* mosquitoes. Polyester nets were impregnated with 1 mL of concentrated *Bacillus* culture supernatant obtained from cultures standardized to 8 McFarland units (∼2.4 × 10^9^ CFU/mL) and allowed to air‐dry before exposure. Positive and negative controls consisted of polyester nets treated with clothianidin 50% WG and distilled water, respectively. Clothianidin was prepared at 1 mg/L (0.001 mg/mL of the commercial formulation), and 1 mL of the solution was applied to the polyester net and allowed to dry. Ten adult *Anopheles* mosquitoes were introduced into each WHO cone fitted with the treated polyester net. Mosquitoes were maintained under the same conditions and provided with a 10% sugar solution during the assay. Each treatment was performed in triplicate following World Health Organization [17] guidelines. Eight *Bacillus* isolates were tested, together with positive and negative controls, resulting in a total of 300 adult mosquitoes: 240 mosquitoes were used for the eight *Bacillus* isolate treatments (30 mosquitoes per isolate), and 60 mosquitoes were allocated to the positive and negative controls (30 mosquitoes per control) (Supporting file: Figure S4). Mortality was recorded after 24 h of exposure. Percentage mortality was calculated as the number of dead mosquitoes divided by the total number of exposed mosquitoes × 100.

(1) Mortality rate %=No. of insect deathsTotal no. of insects×100.

### 2.12. Determination of Lethal Concentrations (LC_50_ and LC_95_)

*Bacillus* isolates that produced at least 50% larval mortality in preliminary assays were further evaluated following the WHO protocol [17]. LC_50_ and LC_95_ of the culture supernatants were determined using third‐instar *Anopheles* mosquito larvae. Culture supernatants were prepared from bacterial cultures standardized to 2, 4, and 8 McFarland units before centrifugation and separation of the cell‐free supernatant fraction. Each assay was conducted in triplicate using 10 third‐instar larvae exposed in 10 mL of distilled water. Larval mortality was recorded at 12, 24, and 48 h after exposure. Negative controls containing distilled water without bacterial material were included. Mortality data from treatments producing 10% to approximately 95% mortality were used for probit analysis to estimate LC_50_ and LC_95_ values at each exposure time.

### 2.13. SDS‐PAGE Analysis

Extracellular proteins from *Bacillus* strains AW1, AW18, AW23, AW29, WS8, WI7, WI45, and WI51 were analyzed using SDS‐PAGE as described by Laemmli [19]. Each strain was cultured in Nutrient Broth at 30°C with shaking at 200 rpm for 24 h. The cultures were centrifuged at 10,000 × g for 10 min at 4°C, and the resulting supernatants were filtered through 0.22‐μm membranes to remove any residual cells. The proteins in the cell‐free supernatants were precipitated by adding trichloroacetic acid (TCA) to a final concentration of 10% (w/v) and incubating on ice for 1 h. The precipitated proteins were collected by centrifugation at 14,000 × g for 20 min at 4°C, washed twice with cold acetone, air‐dried, and resuspended in 2 × Laemmli sample buffer. The samples were boiled for 5 min and loaded (10–20 μL) onto SDS‐polyacrylamide gels along with a prestained molecular marker. Electrophoresis was carried out at 120 V until the dye front reached the bottom of the gel. The gels were stained with Coomassie Brilliant Blue R‐250, destained, and photographed. The protein band patterns were compared among the isolates, and the molecular weights were estimated relative to the standard marker.

### 2.14. Data Analysis

All bioassays were conducted in three independent biological replicates, each consisting of 10 mosquitoes. Percentage mortality was calculated as the number of dead mosquitoes divided by the total number of exposed mosquitoes × 100, and results are presented as mean ± SE. Prior to analysis, the assumptions of normality and homogeneity of variance were assessed and met. Treatment effects were analyzed using one‐way analysis of variance (ANOVA) followed by Tukey’s honestly significant difference (HSD) test in R software (Version 4.3.2). Probit analysis was performed using IBM SPSS Statistics 24 to estimate LC_50_, LC_95_, 95% confidence intervals (CIs), slopes, and SEs according to Finney [20]. Statistical significance was set at *p* ≤ 0.05.

## 3. Results

### 3.1. Characteristics of *Bacillus* Isolates

**FIGURE 1:**
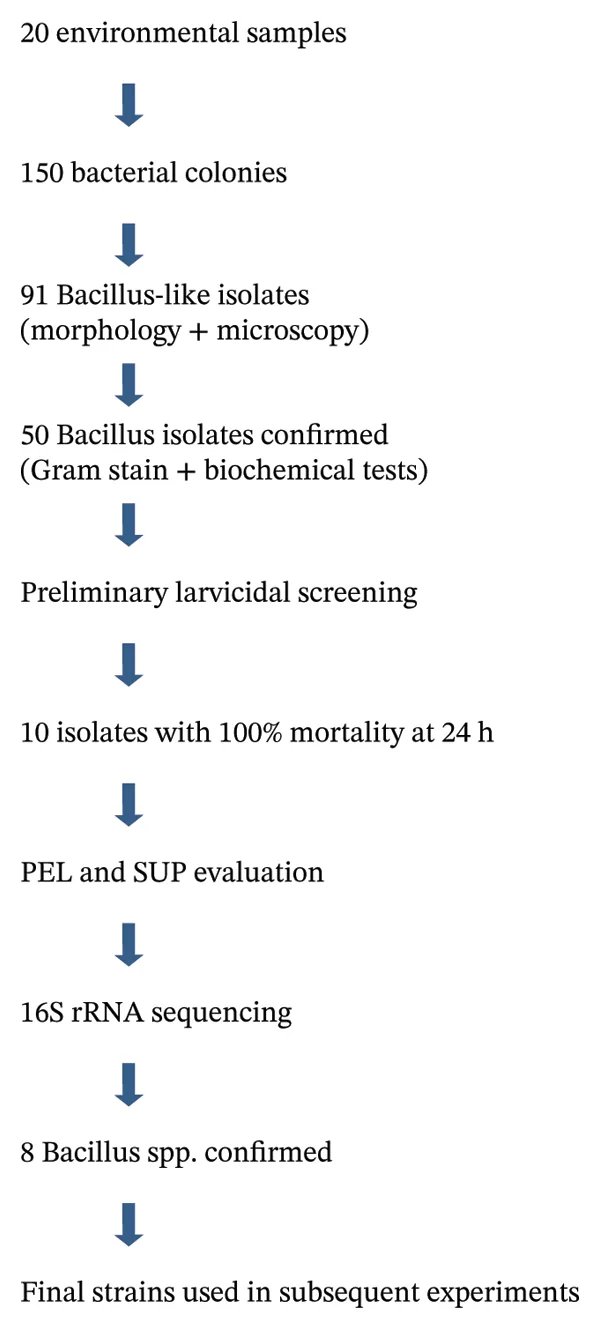
Study flow diagram showing the isolation, screening, molecular characterization, and final selection of 8 *Bacillus* strains from environmental samples.

A total of 20 environmental samples were collected, from which 150 bacterial colonies were isolated. Of these, 91 isolates showed *Bacillus*‐like characteristics based on colony morphology, Gram reaction, and microscopic examination. These presumptive *Bacillus* isolates were obtained from different sources, including soil and sludge (31 isolates), water samples (35 isolates), and dead mosquito samples (25 isolates) (Table 1). The overall workflow from sample collection, bacterial isolation, screening, biochemical characterization, molecular identification, and final selection of 8 active *Bacillus* isolates is presented in Figure 1.

**TABLE 1:** *Bacillus*‐like bacterial colonies isolated from the samples collected from four locations in Ethiopia.

Sample code	Sampling location	Sample type	Number of *Bacillus*‐like isolates
AW	Adami‐Tulu insecticide factory	Soil and sludge	31
WS	Wondo Genet	Spring water	20
SW	Sodere Lake (Bishoftu)	Spring water	15
WI	Afar	Dead mosquito	25
Total			91

**FIGURE 2:**
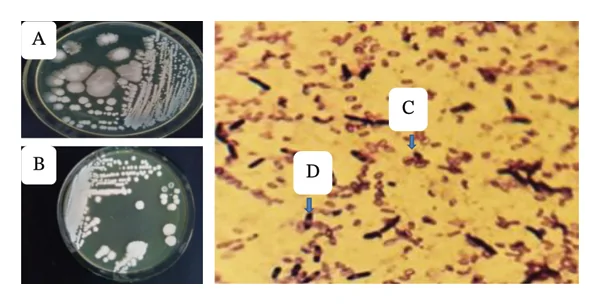
Representative morphological characteristics of *Bacillus*‐like isolates. (A–B) Representative colonies of a *Bacillus*‐like isolate grown on nutrient agar, showing white, glossy, nearly circular colonies with raised centers and slightly irregular margins. (C) Gram‐stained *Bacillus*‐like isolate observed under a compound bright‐field microscope (1000× magnification), showing Gram‐positive rod‐shaped cells with endospores. (D) Arrow indicating an endospore within a vegetative cell.

The presumptive *Bacillus*‐like isolates exhibited diverse colony morphologies; however, most produced white, glossy, nearly circular colonies with raised centers and slightly irregular margins. Microscopic examination showed that these isolates were rod‐shaped, Gram‐positive, and endospore‐forming (Figure 2A–D). Following biochemical characterization, 50 of the 91 presumptive *Bacillus*‐like isolates were confirmed as *Bacillus*‐like isolates consistent with *Bacillus* spp. These isolates were uniformly catalase‐positive, oxidase‐positive, indole‐negative, and capable of hydrolyzing starch, consistent with the characteristics of spore‐forming *Bacillus* species.

### 3.2. Larvicidal Activity

**FIGURE 3:**
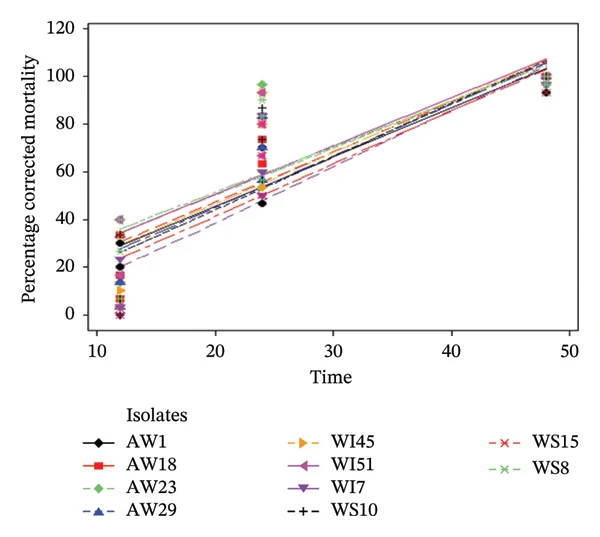
Larvicidal activity of bacterial isolates against *Anopheles* mosquito larvae over time. Percentage‐corrected larval mortality following exposure to bacterial supernatants from 10 bacterial isolates (AW1, AW18, AW23, AW29, WI45, WI51, WI7, WS10, WS15, and WS8) standardized to 8 McFarland units (∼2.4 × 10^9^ CFU/mL). Mortality was assessed after 12, 24, and 48 h of exposure. Values represent the mean mortality percentage from three independent replicates.

**FIGURE 4:**
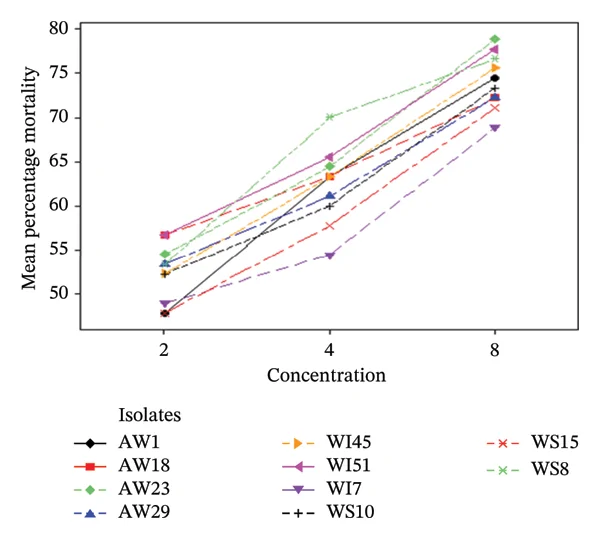
Larvicidal activity of bacterial isolates against third‐instar *Anopheles* mosquito larvae. Mean percentage‐corrected mortality of larvae (*n* = 10 per replicate) exposed to bacterial isolates (AW1, AW18, AW23, AW29, WI45, WI51, WI7, WS10, WS15, and WS8) at 2, 4, and 8 McFarland units. Mortality was assessed after exposure, and values represent the mean of three replicates calculated using Abbott’s formula. The isolates showed concentration‐dependent larvicidal activity.

Based on preliminary screening for larvicidal activity, 50 *Bacillus*‐like isolates exhibited larvicidal activity. These isolates caused more than 50% larval mortality within 24 h, and 100% mortality within 48 h. From these, 10 isolates that achieved 100% larval mortality within 24 h were selected for further evaluation of the larvicidal activities of the bacterial PEL and culture SUP. The PEL fraction produced less than 50% larval mortality, whereas the SUP fraction caused 100% larval mortality within 24 h when prepared from cultures standardized to 8 McFarland units. Supernatants prepared from cultures standardized to 2 and 4 McFarland units produced more than 50% larval mortality within 48 h (Figure 3). The 10 selected isolates were subsequently evaluated at three concentrations. Larvicidal activity increased with increasing concentration, demonstrating a clear concentration‐dependent effect (Figure 4).

### 3.3. Identification of the Bacterial Isolates

**FIGURE 5:**
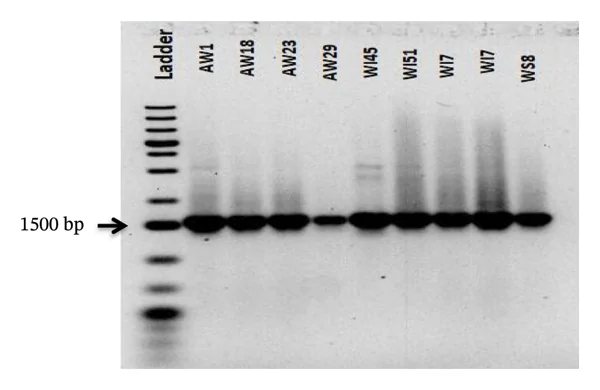
Agarose gel electrophoresis of PCR‐amplified 16S rRNA gene fragments from bacterial isolates. PCR products from bacterial isolates AW1, AW18, AW23, AW29, WI45, WI51, WI7, and WS8 were separated on a 1% agarose gel. Lane M: 1‐kb DNA ladder. All isolates produced a single amplicon of the expected size (∼1500 bp), confirming successful amplification for subsequent sequencing and molecular identification.

Isolates showing the highest biocidal activity were subjected to amplification and sequencing of their 16S rRNA genes, and the resulting sequences were compared with those in the NCBI database. All bacterial isolates produced a single PCR‐amplified fragment of approximately 1500 bp, corresponding to the expected length of the bacterial 16S rRNA gene (Figure 5). Table 2 summarizes the alignment of the 8 bacterial isolates to their closest type strains along with their accession numbers and final designations based on taxonomic thresholds. The NCBI BLASTn results indicated that six isolates belong to the genus *Bacillus*, while two isolates were identified within the genus *Geobacillus*.

**TABLE 2:** Phylogenetic neighbors of *Bacillus* isolates based on similarity to the partial 16S rRNA sequence.

No.	Isolate ID	Closest type strain	Accession number	Percent similarity (%)	Final designation
1	WI45	*Bacillus velezensis* strain 11	PQ780306.1	100	*Bacillus velezensis*
2	WI51	*Bacillus safensis* strain BSFL‐SRN04	PX363126.1	100	*Bacillus safensis*
3	WS8	*Bacillus subtilis* strain Z‐12‐20	PP538033.1	97.67	*Bacillus* sp.
4	WI7	*Bacillus axarquiensis* strain BPRIST011	JF414764.1	95.64	*Bacillus* sp. (putative novel sp.)
5	AW18	*Geobacillus* sp. strain Sd17	OR448040.1	98.02	*Geobacillus* sp.
6	AW23	*Bacillus subtilis* strain T2b1	OQ880567.1	99.91	*Bacillus subtilis*
7	AW1	*Geobacillus subterraneus* strain SH2	MT899189.1	98.06	*Geobacillus* sp.
8	AW29	*Bacillus* sp. (in: *Firmicutes)* strain YIM B13435	PQ608388.1	97.00	*Bacillus* sp.

Strains WI45 and WI51 showed 100% similarity to *Bacillus velezensis strain 11* and *Bacillus safensis strain BSFL-SRN04*, respectively. Isolate AW23 also displayed high sequence identity (99.91%) to *Bacillus subtilis* strain T2b1. Consequently, these three isolates were assigned high‐confidence species‐level identifications. In contrast, the remaining five isolates exhibited sequence similarities ranging from 95.64% to 98.06%, falling below the standard 98.7% threshold recommended for definitive species‐level assignment. To maintain taxonomic caution, these five isolates were designated strictly at the genus level as *Bacillus* sp. (WS8, WI7, and AW29) or *Geobacillus* sp. (AW18 and AW1). Notably, isolate WI7 exhibited a highly divergent similarity score of only 95.64% to its closest match, *Bacillus axarquiensis* strain BPRIST011, highlighting its potential status as a novel phylotype.

**FIGURE 6:**
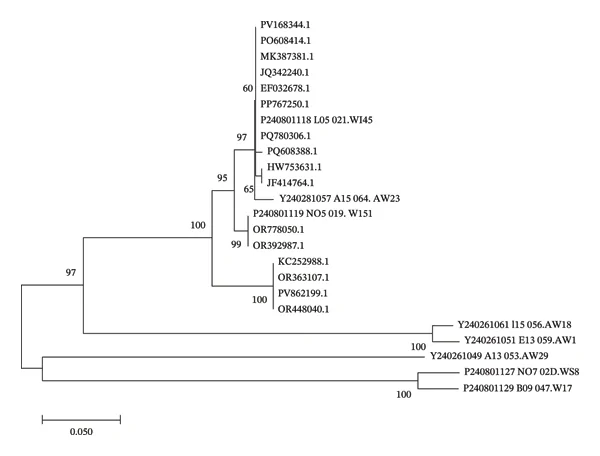
Phylogenetic neighbors of isolates based on similarity to the 16S rRNA sequence. The evolutionary history was inferred using the neighbor‐joining method. The optimal tree with a sum of branch lengths = 1.033 is shown. The tree is drawn to scale, with branch lengths in the same units as those of the evolutionary distances used to infer the phylogenetic tree. The evolutionary distances were computed using the *p*‐distance method and are in the units of the number of base differences per site. The analytical procedure encompassed 24 nucleotide sequences. The complete deletion option was applied to eliminate positions containing gaps and missing data, resulting in a final data set comprising 224 positions. Evolutionary analyses were conducted in MEGA12 utilizing up to 8 parallel computing threads.

Phylogenetic analysis further supported these relationships (Figure 6). Isolate WI51 clustered closely with its reference sequence with a strong bootstrap support of 95%, confirming its close relationship to *Bacillus safensis*. Similarly, isolate WI45 grouped closely with its respective reference sequences, supported by a robust bootstrap value of 97%, validating its identification as *Bacillus velezensis*. Furthermore, isolates WS8, WI7, AW18, AW1, and AW29 all clustered into distinct, stable clades with their respective reference matches, supported by 100% bootstrap values. While these high bootstrap values confirm robust branch topology and clear evolutionary relationships within their respective genera, their underlying sequence divergence necessitates keeping their final assignments restricted to the genus level (*Bacillus* sp. and *Geobacillus* sp.) pending further polyphasic taxonomic validation.

### 3.4. Optimization of Growth Conditions

**FIGURE 7:**
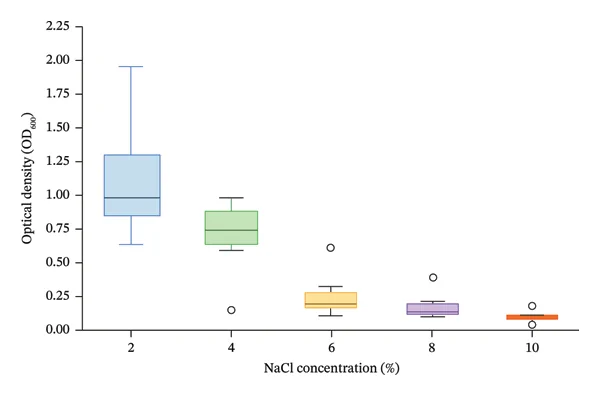
This box plot illustrates how the optical density (OD) mean values for bacterial isolates vary across different salt concentrations. The trend shows a decrease in OD as the salt concentration increases, indicating potential salt‐induced stress.

**FIGURE 8:**
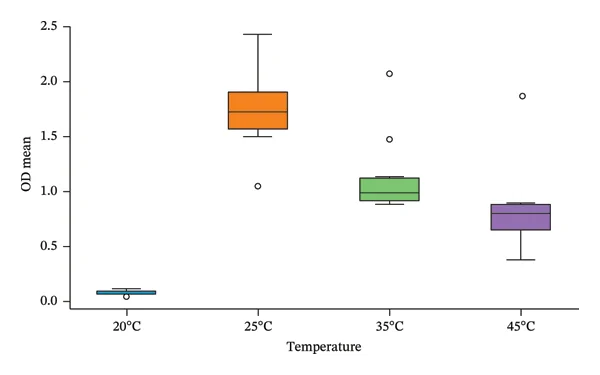
This box plot illustrates the average optical density (OD) values recorded after 24 h of incubation at 4 distinct incubation temperatures (20°C, 25°C, 35°C, 45°C).

**FIGURE 9:**
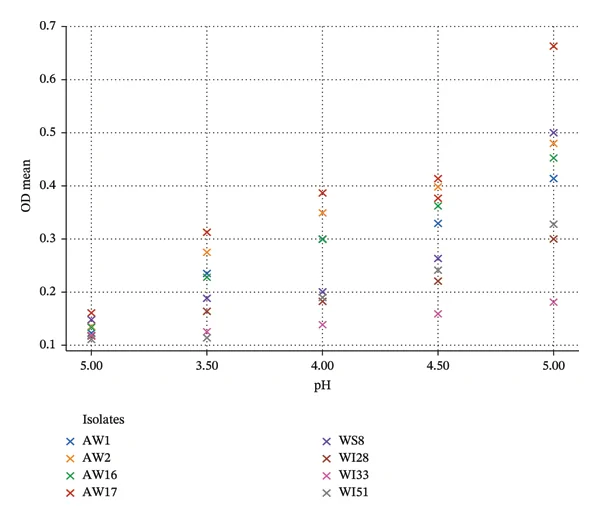
Optical density (OD) of bacterial isolates after 24 h incubation at different pH values. Growth increased from pH 3.5 to 5.0, with maximum OD at pH 5.0 and minimum OD at pH 3.0, indicating that slightly acidic conditions favored bacterial growth.

The growth conditions of all the strains were optimized for pH, NaCl tolerance, and temperature. NaCl tolerance was checked, with most strains growing best in the range of 2%–6% (Figure 7). Similarly, growth was assessed at temperatures ranging from 25°C to 45°C, with 25°C being the optimal temperature (Figure 8). From the results, it was concluded that almost all bacterial strains grow under acidic conditions at pH 3, 3.5, and 4 (Figure 9). The 8 selected bacterial strains in this study were isolated from hot spring lakes with varying salinity, soil, and factory sludges exposed to different chemical insecticides and pH levels. To survive in such an environment, *Bacillus* strains may be forced to adapt and produce traits that make them promising biocontrol agents against mosquitoes.

### 3.5. Adulticidal Activity

**FIGURE 10:**
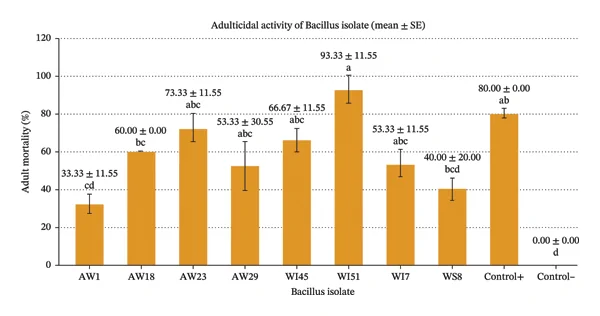
Adulticidal activity of *Bacillus* strains against adult mosquitoes. Data are presented as mean ± standard error (SE; *n* = 3 biological replicates), with 10 adult mosquitoes per replicate. Bars marked with the same lowercase letter do not differ significantly according to one‐way analysis of variance (ANOVA) followed by Tukey’s HSD test (*p* < 0.05). Positive control: clothianidin; negative control: distilled water.

Eight *Bacillus* isolates exhibiting high larvicidal activity were selected for adulticidal bioassays. A total of 240 five‐day‐old adult *Anopheles* mosquitoes were used to evaluate the 8 isolates, with 10 mosquitoes per replicate and three biological replicates per isolate. Positive and negative control groups were tested under the same experimental conditions. Among the tested isolates, WI51 exhibited the highest adulticidal activity, with a mean mortality of 93.33 ± 11.55%, followed by the positive control (80.00 ± 0.00%), AW23 (73.33 ± 11.55%), and WI45 (66.67 ± 11.55%). The mortality produced by WI51 exceeded that of the Clothianidin positive control at the concentration tested, whereas no mortality was observed in the negative control (Figure 10).

### 3.6. LC_50_ and LC_95_

The LC_50_ and LC_95_ values ranged from 2.47 (0.87–5.88) to 3.49 (1.45–6.67) McF and 5.40 (3.33–7.84) to 23.38 (21.53–27.92) McF, respectively, after 12 h of exposure. After 24 h of exposure, the LC_50_ values ranged from 0.24 (0.01–0.65) to 0.56 (0.03–0.89) McF, while the LC_95_ values ranged from 1.57 (0.55–4.34) to 4.14 (2.20–6.80) McF (Table 3). Complete (100%) larval mortality was observed after 48 h of exposure.

**TABLE 3:** Toxicity of *Bacillus* isolates against third‐instar larvae of *Anopheles* mosquitoes.

Strain	12 h			24 h		
	LC_50_ (95% CI) (McF)	LC_95_ (95% CI) (McF)	Slope (±SE)	LC_50_ (95% CI) (McF)	LC_95_ (95% CI) (McF)	Slope (±SE)
AW1	3.36 (2.21–5.67)	15.02 (12.31–17.90)	1.26 (± 0.30)	0.56 (0.03–0.89)	1.80 (1.08–5.20)	2.53 (± 0.63)
AW18	3.49 (1.45–6.67)	18.22 (14.37–20.82)	1.19 (± 0.29)	0.29 (0.02–0.69)	4.14 (2.20–6.80)	1.03 (± 0.64)
AW23	3.41 (1.43–6.61)	23.38 (21.53–27.92)	1.08 (± 0.26)	0.47 (0.01–2.81)	1.80 (1.0–5.20)	1.11 (± 0.27)
AW29	3.05 (1.28–5.56)	10.91 (6.22–12.45)	1.33 (± 0.32)	0.38 (0.01–1.70)	3.57 (1.54–6.11)	1.32 (± 0.55)
WS8	2.49 (0.78–4.40)	11.32 (9.67–15.07)	1.13 (± 0.27)	0.38 (0.07–1.87)	1.75 (0.67–4.56)	1.94 (± 0.80)
WI7	3.07 (1.86–6.09)	6.59 (4.21–9.01)	2.63 (± 0.69)	0.55 (0.08–1.89)	3.67 (1.88–7.76)	1.56 (± 0.39)
WI45	3.07 (1.57–5.98)	10.24 (7.68–13.0)	1.39 (± 0.34)	0.45 (0.01–1.76)	1.63 (0.93–8.31)	2.37 (± 0.81)
WI51	2.47 (0.87–5.88)	5.40 (3.33–7.84)	2.10 (± 0.53)	0.24 (0.01–0.65)	1.57 (0.55–4.34)	1.71 (± 0.91)

*Note:* LC_50_ and LC_95_ represent the concentrations required to kill 50% and 95% of the third‐instar *Anopheles* larval population, respectively. Values were estimated by probit analysis and are presented with 95% confidence intervals (CIs) and slope ± standard error (SE). Each bioassay consisted of three independent biological replicates, with 10 larvae per replicate. LC values are expressed as McFarland units (McF) and were determined at 12 and 24 h posttreatment.

Eight highly active bacterial isolates were selected based on their mosquitocidal activity and were identified by 16S rRNA gene sequencing. All isolates were classified to the genus level as *Bacillus* sp. or *Geobacillus* sp., with sequence accession numbers deposited in the NCBI GenBank database. The isolates originated from different environmental sources, including insecticide factory effluent, spring water, and dead mosquito samples collected from different locations. The identified strains and their observed biological activities are summarized in Table 4.

**TABLE 4:** Summary of source environment and biological activity profile of selected *Bacillus* isolates.

Strain	Source environment	Collection site	Observed biological activity
AW1	Factory sludge (effluent)	Adami‐Tulu insecticide factory	Larvicidal and adulticidal activity
AW18	Factory sludge (effluent)	Adami‐Tulu insecticide factory	Larvicidal and adulticidal activity
AW23	Factory sludge (effluent)	Adami‐Tulu insecticide factory	High larvicidal and adulticidal activity
AW29	Factory sludge (effluent)	Adami‐Tulu insecticide factory	Larvicidal and adulticidal activity
WS8	Spring water	Wondogenet	Larvicidal and adulticidal activity
WI7	Dead mosquito	Afar	Larvicidal and adulticidal activity
WI45	Dead mosquito	Afar	High larvicidal and adulticidal activity
WI51	Dead mosquito	Afar	High larvicidal and adulticidal activity

### 3.7. SDS‐PAGE Profiling

**FIGURE 11:**
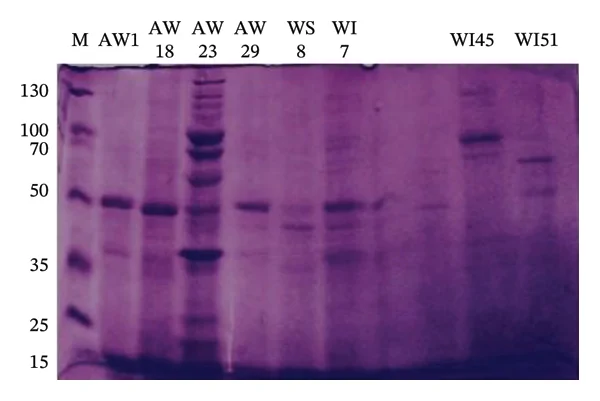
SDS‐PAGE protein profiles of bacterial isolates. Protein banding patterns of bacterial isolates AW1, AW18, AW23, AW29, WS8, WI7, WI45, and WI51 were analyzed by SDS‐PAGE. The molecular weight marker (M) is shown on the left, with protein sizes indicated in kDa. Distinct protein bands observed among the isolates demonstrate variations in their protein profiles.

SDS‐PAGE analysis of extracellular proteins produced by the *Bacillus* isolates revealed distinct protein banding patterns ranging from approximately 15–130 kDa. Prominent protein bands of approximately 60–70 kDa were observed in isolates AW23, AW29, WI45, and WI51. These bands are consistent with the molecular weight range reported for putative Cry/Cyt‐like proteins previously associated with mosquitocidal *Bacillus* spp., although their identities were not confirmed in the present study. In addition, faint protein bands of approximately 25–30 kDa were detected in some of these isolates, which are consistent with the molecular weight range of putative Cyt‐like proteins. In contrast, isolates AW1, AW18, WS8, and WI7 exhibited faint or no detectable protein bands within these molecular weight ranges. These findings indicate differences in extracellular protein profiles among the isolates and suggest that AW23, AW29, WI45, and WI51 may produce proteins associated with enhanced mosquitocidal activity (Figure 11).

## 4. Discussion

Despite decades of control efforts, the growing resistance of mosquito vectors to chemical insecticides and the limited availability of effective antimalarial drugs have contributed to persistently high mortality rates [21]. Given this growing issue of mosquito resistance to chemical insecticides, malaria continues to be a leading cause of mortality in Ethiopia [22]. Therefore, this research aims to develop and implement eco‐friendly biocidal resources as alternative control measures against mosquito vectors, thereby reducing the environmental impact of chemical insecticides.

*Bacillus* species are well recognized for their mosquitocidal activity, with previous studies demonstrating that *Bacillus thuringiensis* and *Bacillus subtilis* can cause substantial larval mortality within 24 h [23]. In the present study, 150 bacterial colonies were isolated from diverse environmental sources, including factory sludge (effluent), soil, spring water, and dead mosquitoes. Following morphological, microscopic, and biochemical characterization, 50 *Bacillus*‐like isolates were selected, of which 8 highly active isolates were subjected to further characterization. The selected isolates originated from environmentally diverse habitats with temperatures ranging from approximately 30°C–93.4°C and pH values between 3.5 and 8.5 [24]. Consistent with their environmental origin, the isolates grew under acidic conditions (pH 3–5), tolerated 2%–6% NaCl, and exhibited growth between 25°C and 45°C. Similar habitats have been reported as important reservoirs of entomopathogenic *Bacillus* species with biocontrol potential [8, 25, 26]. The broad environmental tolerance of these isolates may enhance their ecological fitness and support their potential application as biological control agents against mosquito vectors.

Entomopathogenic *Bacillus* strains, including *Bacillus thuringiensis* [27], *Bacillus subtilis*, *B. thuringiensis subsp. israelensis* [28], *Bacillus cereus*, *Bacillus pumilus* [8], *Brevibacillus laterosporus*, and *Paenibacillus polymyxa* [25], have been widely recognized as effective biocontrol agents against mosquito larvae and adults. Consistent with these previous reports, the present study identified eight promising *Bacillus* isolates, and their culture SUP fractions demonstrated considerable larvicidal activity against *Anopheles* mosquitoes. The SUP fractions obtained from bacterial suspensions standardized at 8 McFarland units (∼2.4 × 10^9^ CFU/mL) resulted in 100% mortality within 24 h, whereas SUP fractions derived from lower bacterial concentrations of 2 McFarland units (∼6 × 10^8^ CFU/mL) and 4 McFarland units (∼1.2 × 10^9^ CFU/mL) caused more than 50% mortality within 48 h.

The relatively low occurrence rate of mosquitocidal activity may be attributed to the diversity of *Bacillus* strains [29]. Nevertheless, the strains showed mosquitocidal activity with varying levels of potency. The larvicidal activities detected in the culture medium and supernatant suggest that secreted molecules in the supernatant were more active than the cellular components found in the pellets [30, 31]. The higher activity observed in the supernatant fraction suggests that extracellular metabolites or soluble insecticidal compounds may contribute to mosquito mortality; however, the specific active components require further investigation. Although only a small difference was observed between the effects induced by the supernatant fractions, insecticidal activity increased with higher concentrations of bacterial products [32]. The observed parasporal inclusion bodies and crystal morphologies were consistent with characteristics commonly reported for entomopathogenic *Bacillus* strains by Loutfi et al. [33].

The 8 selected larvicidal *Bacillus*‐like isolates exhibited similar morphological and biochemical characteristics, including Gram‐positive rod‐shaped cells, endospore formation, and typical *Bacillus*‐associated biochemical profiles, consistent with previous reports on mosquito‐active *Bacillus* strains [34, 35]. However, the variation in LC_50_ and LC_95_ values among isolates indicates that similar phenotypic characteristics do not necessarily correspond to equivalent insecticidal activity, as reported for entomopathogenic *Bacillus* strains [36]. All selected isolates showed activity against both larval and adult mosquito stages, with WI51 demonstrating the highest mosquitocidal activity, comparable to previously described dual‐stage active *Bacillus* isolates [37]. The higher activity of the WI51 culture supernatant suggests that extracellular metabolites may contribute to its insecticidal effect, supporting its potential for further evaluation as a biological mosquito control agent [38].

In our results, the highest mortality of adult mosquitoes was observed compared to previous studies. The observed adult mortality within 24 h may be associated with the production of insecticidal metabolites by the *Bacillus* isolates; however, the specific mechanisms responsible for adult toxicity require further investigation. Differences in susceptibility between mosquito developmental stages may be related to physiological differences between larvae and adults, although the mechanisms underlying adult susceptibility remain unclear [39]. The insecticidal activity may also be due to the bacterial strains adapted to produce compounds with characteristics similar to chemical insecticides [32, 40].

The 16S rRNA gene sequence has been widely used for the identification of unknown bacteria at the genus or species level [41, 42]. In the present study, phylogenetic analysis based on 16S rRNA sequences revealed that all eight isolates were closely related to known *Bacillus* type strains, confirming their classification within the genus. Sequence similarity with the closest type strains ranged from 95.64% to 100%, indicating close relationships with known *Bacillus* species. Phylogenetic analysis further demonstrated that the isolates formed distinct clusters with known *Bacillus* strains, reflecting clear evolutionary relationships. The separation into different clades indicates genetic diversity that may be associated with their environmental origins and functional traits, including insecticidal activity [43, 44]. The origin of the AW isolates from pesticide factory environments suggests that these strains were isolated from chemically influenced habitats. However, whether this environmental origin contributes to their insecticidal activity requires further investigation [45].

Isolates from dead insects (WI group) suggest a potential role as natural entomopathogenic or biocontrol agents, possibly contributing to insect mortality through toxin production or other virulence factors [46–48]. The isolate from spring water (WS group) reflects its environmental origin and adaptation to aquatic habitats, suggesting that these strains are part of the natural microbial community in water ecosystems and may possess beneficial ecological or biocontrol properties [49].

Among the 8 *Bacillus* strains, the WI group demonstrated the highest larvicidal and adulticidal activities, followed by the AW group, while the WS strain showed comparatively lower activity, suggesting that insect‐associated isolates may possess enhanced mosquitocidal potential. Notably, strain WI51, belonging to the WI group, exhibited the highest activity among all strains, further supporting the potential of insect‐associated *Bacillus* strains as effective biocontrol agents.

This study was limited to bioassays of bacterial culture supernatant and pellet preparations. Toxicity to other microorganisms and ecotoxicology of the preparation were not assessed [50]. Further studies on the activity of the preparation at different temperatures to study shelf life and efficacy under field conditions are required before considering their application as a biocidal agent for vector control [51].

SDS‐PAGE analysis revealed protein profiles ranging from approximately 15–130 kDa among the *Bacillus* isolates. Several isolates showed bands within molecular weight ranges commonly associated with Cry (∼60–70 kDa) and Cyt (∼25–30 kDa) proteins, suggesting the possible presence of putative Cry/Cyt‐like proteins. Similar protein profiles have been reported among mosquitocidal *Bacillus* strains; however, SDS‐PAGE alone cannot confirm toxin identity, and further analyses such as PCR, immunological assays, or proteomic characterization are required for confirmation [52, 53].

Isolates AW23, AW29, WI45, and WI51 exhibited prominent bands around 130, 65–70, and 27 kDa, which fall within molecular weight ranges previously associated with Cry/Cyt‐like proteins. The higher abundance of these protein bands may contribute to the observed larvicidal activity, although their specific roles require further validation [54, 55]. In contrast, weak or undetectable bands in AW1, AW18, WS8, and WI7 may reflect differences in protein abundance or production under the tested conditions rather than the absence of toxin‐related genes [56]. Variation in protein profiles among isolates may contribute to differences in larvicidal activity, as reported for mosquitocidal *Bacillus* strains [57–59]. Overall, AW23, AW29, WI45, and WI51 showed promising putative Cry/Cyt‐like protein profiles and warrant further characterization as potential biological control agents against *Anopheles* mosquitoes [60].

## 5. Conclusions

This study demonstrated that *Bacillus* strains isolated from insecticide factory effluent and other environmental sources exhibited significant larvicidal and adulticidal activity against *Anopheles* mosquitoes under laboratory conditions. In total, 8 isolates were identified via 16S rRNA gene sequencing and showed high larvicidal and adulticidal activity against both larval and adult life stages, with the highest mortality observed within 24 h at a concentration of 8 McFarland units. SDS‐PAGE analysis revealed protein bands within molecular weight ranges commonly associated with putative Cry/Cyt‐like proteins in selected strains, suggesting the possible involvement of toxin‐associated proteins in their mosquitocidal activity; however, further molecular and biochemical analyses are required to confirm their identity and functional roles. Overall, the selected *Bacillus* strains represent promising candidates for further investigation as biological control agents. Additional studies are needed to evaluate their stability, shelf life, safety, and efficacy under semifield and field conditions before their potential application in mosquito control programs.

## Author Contributions

Musin Kelel: writing–original draft, conceptualization, supervision, methodology, formal analysis, data curation, visualization, validation, writing–review and editing, software. Tirsit Tibebu: writing–original draft, conceptualization, investigation, methodology, formal analysis, data curation, visualization, validation, software. Mesfin Tafesse: conceptualization, supervision, methodology, visualization, validation, writing–review and editing. Mesfin Getachew Tadesse: conceptualization, investigation, project administration, resources, methodology, visualization, validation. Gemechu Tafa: conceptualization, investigation, methodology, data curation, validation, visualization.

## Funding

The research funding was provided by AASTU under the university–industry linkage collaboration program.

## Disclosure

All authors have read and agreed to the published version of the manuscript.

## Ethics Statement

The authors have nothing to report.

## Consent

The authors have nothing to report.

## Conflicts of Interest

The authors declare no conflicts of interest.

## Supporting Information

Additional supporting information can be found online in the Supporting Information section.

## Supporting information


**Supporting Information** All Supporting Information provided with this article support and extend the main findings presented in the manuscript. Figure S1 illustrates the sampling locations and source materials: (A) waste effluent sludge collected from insecticide production facilities; (B) varied sludge types reflecting different insecticide manufacturing processes; and (C) natural mosquito breeding sites at Sodere (Bishoftu), from which water and dead Anopheles larvae were collected. Figure S2 documents the bioassay experimental setup: (A) bacterial supernatant preparations standardized to 2, 4, and 8 McFarland units; (B) labeled assay wells containing 10 mL of water; and (C) larval exposure assays with 10 Anopheles larvae per well across three biological replicates. Figure S3 shows complete (100%) larval mortality observed within 24 h of exposure. Figure S4 details the adulticidal bioassay methods: (A) cup‐based assays using treated, air‐dried filter papers; and (B) standard WHO cone assays using impregnated polyester netting, with sugar provision for surviving adults. Table S1 presents full percentage‐corrected larval mortality data across all three concentrations and exposure time points, calculated using Abbott’s formula. All supplementary files are available alongside the online version of this article.

## Data Availability

All data generated or analyzed during this study are included in this published article and its supporting information files.
