# Supplementary material for: Native Bacillus Strains Crude Culture Product Exhibits High Biocidal Activities Against Larvae and Adult Anopheles Mosquitoes
Source: J Trop Med. 2026 Aug 27;2026:8822832. doi: 10.1155/jotm/8822832 (PMC13519662; doi:10.1155/jotm/8822832)
Supplement: Supplementary file 1 [file JOTM-2026-8822832-s001.zip › Supplimentary file-1.docx]

**C**

**B**

**A**

**Supplementary file: Figure S1**. Samples were collected from different locations. (A) Waste effluents (sludge) that had collected from different types of chemical insecticide production over this treatment plant. (B) Different types of sludge in color and content that are released according to the type of insecticide (C) Sodere (Bishoftu) mosquito breeding area that water and dead *Anopheles* mosquito larvae
