# Supplementary material for: Native Bacillus Strains Crude Culture Product Exhibits High Biocidal Activities Against Larvae and Adult Anopheles Mosquitoes
Source: J Trop Med. 2026 Aug 27;2026:8822832. doi: 10.1155/jotm/8822832 (PMC13519662; doi:10.1155/jotm/8822832)
Supplement: Supplementary file 1 [file JOTM-2026-8822832-s001.zip › Supplimentary file-2.docx]

were collected.

**C**

**A**

**B**

**Supplementary file: Figure S2.** (A) Bacterial toxin preparations standardized to 2 McFarland units (~6 × 10⁸ CFU/mL), 4 McFarland units (~1.2 × 10⁹ CFU/mL), and 8 McFarland units (~2.4 × 10⁹ CFU/mL) in test tubes. (B) Labelled assay wells containing 10 mL of water. (C) Exposure setup: 10 third‑instar *Anopheles* larvae per well, with 1 mL of bacterial toxin added for each treatment; untreated groups served as controls, with all assays run in three biological replicates.
