# Supplementary material for: Native Bacillus Strains Crude Culture Product Exhibits High Biocidal Activities Against Larvae and Adult Anopheles Mosquitoes
Source: J Trop Med. 2026 Aug 27;2026:8822832. doi: 10.1155/jotm/8822832 (PMC13519662; doi:10.1155/jotm/8822832)
Supplement: Supplementary file 1 [file JOTM-2026-8822832-s001.zip › Supplimentary file-3.docx]

**B**

**A**

**Supplementary file: Figure S3.** (A-B) Mortality of the *Anopheles* mosquito larvae 100% within 24 hrs
