# Supplementary material for: Native Bacillus Strains Crude Culture Product Exhibits High Biocidal Activities Against Larvae and Adult Anopheles Mosquitoes
Source: J Trop Med. 2026 Aug 27;2026:8822832. doi: 10.1155/jotm/8822832 (PMC13519662; doi:10.1155/jotm/8822832)
Supplement: Supplementary file 1 [file JOTM-2026-8822832-s001.zip › Supplimentary FIle-4.docx]

**A**

**B**

**Supplementary file: Figure S4.** Adult mosquito bioassay (A) Cup bioassay, the papers were allowed to air dry, and each was transferred into a labelled cup. The papers were spread to ensure they completely covered the base area of the cone. (B) Cone bioassay, this experiment was also performed using a cone with impregnated and dried polyester net. Dead mosquitoes are laid on the net and the live (resistant) suck sugar on the top of the cotton.
