# Supplementary material for: Native Bacillus Strains Crude Culture Product Exhibits High Biocidal Activities Against Larvae and Adult Anopheles Mosquitoes
Source: J Trop Med. 2026 Aug 27;2026:8822832. doi: 10.1155/jotm/8822832 (PMC13519662; doi:10.1155/jotm/8822832)
Supplement: Supplementary file 1 [file JOTM-2026-8822832-s001.zip › Supplimentary Table-1.docx]

| **Isolates** | **Conc.in CFU/ml** | **Percentage corrected mortality** | | |
| --- | --- | --- | --- | --- |
|  |  | **12hrs** | **24hrs** | **48hrs** |
| **AW1** | 2 units | 3.44 | 46.7 | 93.4 |
|  | 4 units | 20 | 70 | 100 |
|  | 8 units | 30 | 93.4 | 100 |
| **Aw18** | 2 units | 6.7 | 63.4 | 100 |
|  | 4 units | 16.7 | 73.4 | 100 |
|  | 8 units | 33.4 | 83.4 | 100 |
| **AW23** | 2 units | 13.4 | 53.4 | 96.7 |
|  | 4 units | 13.4 | 80 | 100 |
|  | 8 units | 40 | 96.7 | 100 |
| **AW29** | 2 units | 3.44 | 56.7 | 100 |
|  | 4 units | 13.4 | 70 | 100 |
|  | 8 units | 33.4 | 83.4 | 100 |
| **WS8** | 2 units | 6.7 | 56.7 | 96.7 |
|  | 4 units | 26.7 | 83.4 | 100 |
|  | 8 units | 40 | 90 | 100 |
| **WS10** | 2 units | 0 | 56.6 | 100 |
|  | 4 units | 6.7 | 73.4 | 100 |
|  | 8 units | 33.4 | 86.7 | 100 |
| **WS15** | 2 units | 0 | 50 | 93.4 |
|  | 4 units | 6.7 | 66.7 | 100 |
|  | 8 units | 33.4 | 80 | 100 |
| **WI7** | 2 units | 0 | 50 | 96.7 |
|  | 4 units | 3.4 | 60 | 100 |
|  | 8 units | 23.3 | 83.4 | 100 |
| **WI45** | 2 units | 3.4 | 53.4 | 100 |
|  | 4 units | 10 | 80 | 100 |
|  | 8 units | 33.4 | 93.4 | 100 |
| **WI51** | 2 units | 3.4 | 66.7 | 100 |
|  | 4 units | 16.7 | 80 | 100 |
|  | 8 units | 40 | 93.4 | 100 |
| **Control (+) Clothianidin** | Cumulative result | 38 | 80.1 | 100 |
| **Control (-)** | Cumulative result | 0 | 0 | 0 |

**Supplementary file: Table S1.** Percentage corrected mortality of *Anopheles* mosquito larvae at three concentrations of bacteria toxin and three time exposures using Abbott’s formula
